# Supplementary material for: xMD-miRNA-seq to generate near in vivo miRNA expression estimates in colon epithelial cells
Source: Sci Rep. 2018 Jun 28;8:9783. doi: 10.1038/s41598-018-28198-z (PMC6023933; doi:10.1038/s41598-018-28198-z)
Supplement: Supplementary file 1 — Supplemental Figures 1 and 2 [file 41598_2018_28198_MOESM1_ESM.pdf]

xMD-miRNA-seq to generate near *in vivo* miRNA expression estimates in colon epithelial cells

Avi Z. Rosenberg[1†]; Carrie Wright[2*†], Karen Fox-Talbot[1], Anandita Rajpurohit[2], Courtney Williams[2], Corey Porter[1], Olga Kovbasnjuk[3], Matthew N. McCall[4], Joo Heon Shin[2], Marc K. Halushka[1]*

[†] These authors contributed equally to this work.

1. Department of Pathology, Johns Hopkins University SOM, Baltimore MD, 21205, USA.

2. The Lieber Institute for Brain Development, Baltimore MD, 21205, USA.

3. Department of Medicine, Division of Gastroenterology, Johns Hopkins University SOM, Baltimore MD, 212105, USA.

4. Department of Biostatistics and Computational Biology, University of Rochester Medical Center, Rochester, NY 14642, USA.

Supplemental Figures

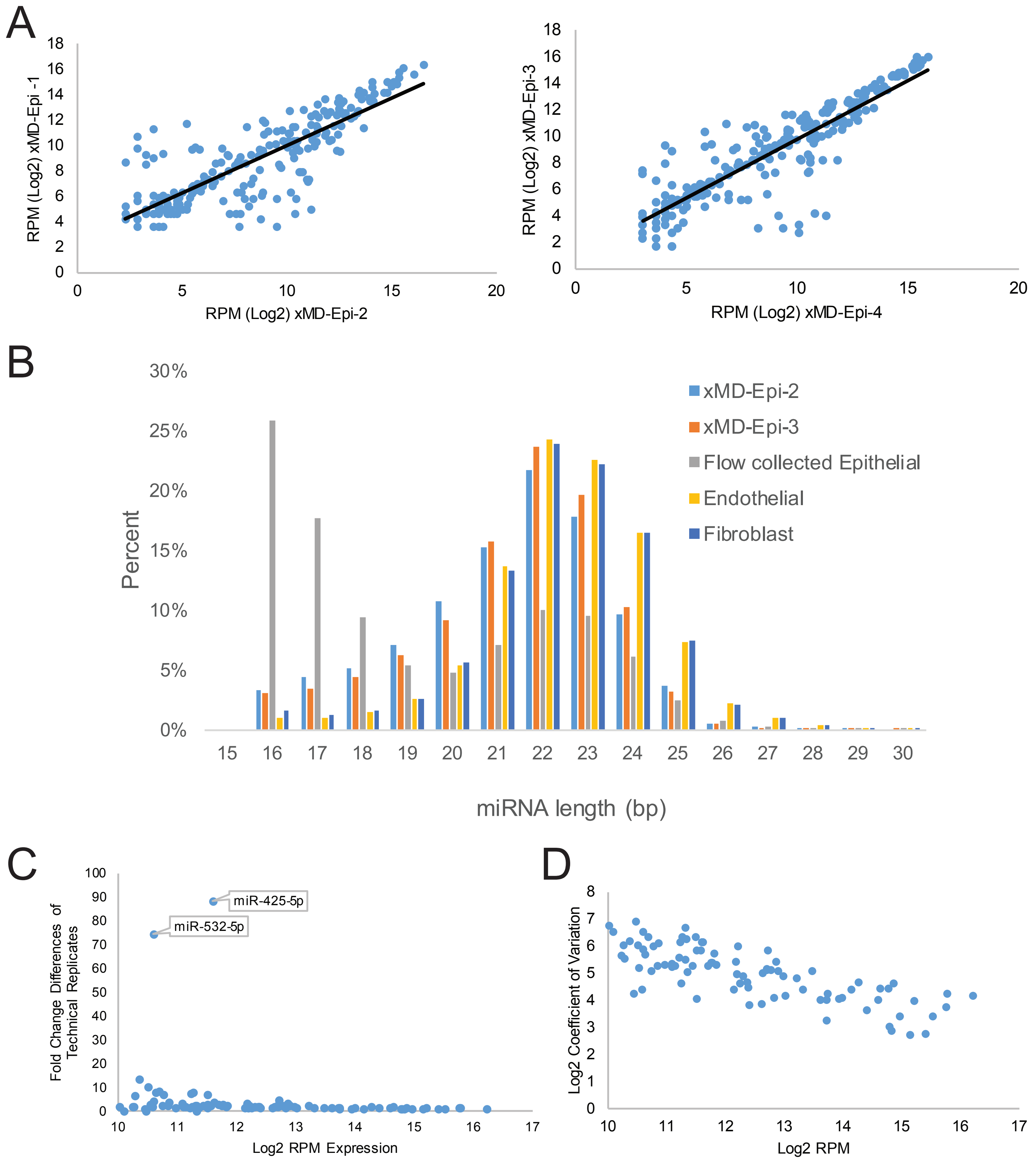

Supplemental Figure 1. A) Pairwise scatter plots of technical replicates of xMD-Epi-1 vs 2 and xMD-Epi-3 vs 4. B) Relative length (in bp) of miRNAs and their isomiRs for 5 samples. The xMD-derived samples are more equivalent to cell culture cell distributions than to the flow collected epithelial cells, which are enriched for shorter sequences. C) A plot of fold change differences between technical replicates of xMD-Epi samples vs average log2 RPM expression showing larger variability occurring with more lowly expressed miRNAs. Two extreme outliers were noted. D) Plot of the coefficient of variation versus log2 RPM expression showing similar overall variation across these 90 miRNA samples.

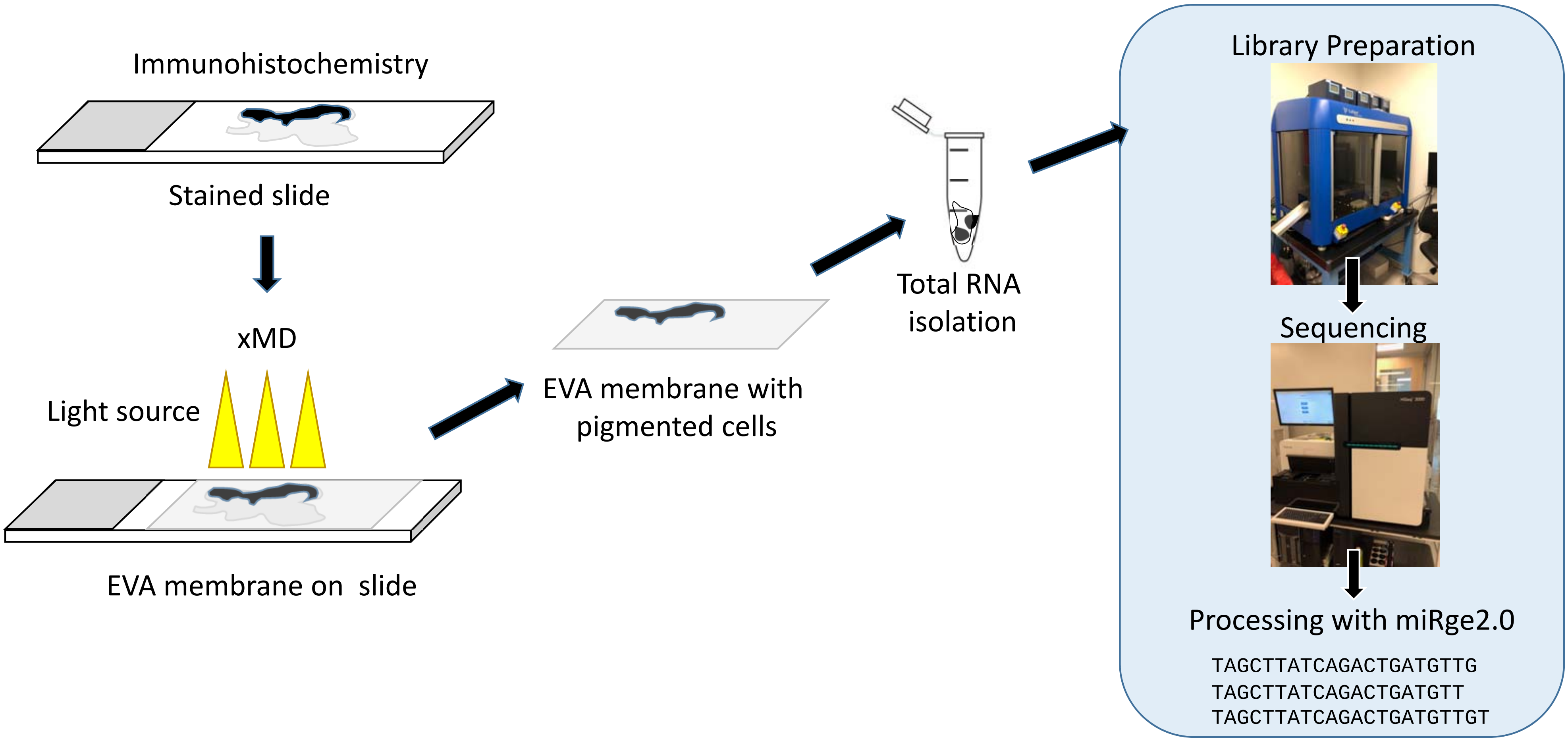

Supplemental Figure 2. The general workflow of xMD-miRNA-seq. Slides are stained through standard immunohistochemistry using RNA precautions. xMD is performed to transfer pigmented cells from the slide to the EVA membrane. The EVA membrane is placed in a tube and the cell material including RNA is eluted. Then a standard sequencing library preparation, run on a high-throughput sequencer and processing of FASTQ files is performed (blue box).
